# Primary and secondary aerenchyma oxygen transportation pathways of *Syzygium kunstleri* (King) Bahadur & R. C. Gaur adventitious roots in hypoxic conditions

**DOI:** 10.1038/s41598-021-84183-z

**Published:** 2021-02-25

**Authors:** Hong-Duck Sou, Masaya Masumori, Takashi Yamanoshita, Takeshi Tange

**Affiliations:** 1 Urban Forest Research Center, National Institute of Forest Science, Seoul, 02455 South Korea; 2 Graduate School of Agricultural and Life Sciences, The University of Tokyo, Yayoi 1-1-1, Bunkyo-ku, Tokyo, 113-8657 Japan

**Keywords:** Ecology, Physiology, Plant sciences

## Abstract

Some plant species develop aerenchyma to avoid anaerobic environments. In *Syzygium kunstleri* (King) Bahadur & R. C. Gaur, both primary and secondary aerenchyma were observed in adventitious roots under hypoxic conditions. We clarified the function of and relationship between primary and secondary aerenchyma. To understand the function of primary and secondary aerenchyma in adventitious roots, we measured changes in primary and secondary aerenchyma partial pressure of oxygen (pO_2_) after injecting nitrogen (N_2_) into the stem 0–3 cm above the water surface using Clark-type oxygen microelectrodes. Following N_2_ injection, a decrease in pO_2_ was observed in the primary aerenchyma, secondary aerenchyma, and rhizosphere. Oxygen concentration in the primary aerenchyma, secondary aerenchyma, and rhizosphere also decreased after the secondary aerenchyma was removed from near the root base. The primary and secondary aerenchyma are involved in oxygen transport, and in adventitious roots, they participate in the longitudinal movement of oxygen from the root base to root tip. As cortex collapse occurs from secondary growth, the secondary aerenchyma may support or replace the primary aerenchyma as the main oxygen transport system under hypoxic conditions.

## Introduction

Flooding is a significant environmental stress for plants growing in peat-swamp forests, while periodic and constant flooding can prevent growth, yield, and distribution of plants^1,2^. Flooding occurs when porous soil is saturated with water due to poor drainage. The rate of oxygen diffusion in water is about 10^4^-fold slower than in air and thus, oxygen transfer in submerged plant roots is limited and aerobic respiration can be inhibited^3^. Oxygen concentration decreases in flooded plant tissues while that of carbon dioxide can increase from microbial and root respiration^4^. This type of oxygen deficiency affects nutrient and water absorption in flooded plants^5,6^ and since roots and rhizomes are aerobic organs, the results can be fatal because, as aerobic respiration halts, levels of energy‐rich adenylates rapidly drops, inducing a significant decline in ion uptake and transport^7–9^.

Plants cannot survive for long periods without oxygen, but some species have developed strategies to avoid anaerobic conditions. In flood-tolerant species, changes in morphological and metabolic levels in tissues help plants adapt to flooding conditions. To overcome the complications caused by flooding, some plant species have a morphological escape mechanism known as low-oxygen escape syndrome, common in plant species that have adapted to prolonged flooding^10^. These phenotypic traits include the formation of aerenchyma, adventitious roots, leaf anatomical modifications, and gas pressurization by the porous tissues^10^. During development, plants can avoid the low-O_2_ stress caused by flooding through multifaceted alterations in cellular and organ structure thereby promoting the diffusion of O_2_^10^.

Aerenchyma is a plant tissue that forms spaces in the leaves, stems, and roots, allowing gas exchange^11^. Aerenchyma can facilitate the movement of gases (O_2_, CO_2_, ethylene, and methane) in and out of tissues, and move oxygen from the stem to the root in plants exposed to flooding conditions which is essential for plant survival since it reduces hypoxic stress^11–13^. The movement of oxygen through the aerenchyma from the stem to the root in plants exposed to flooding conditions is very important for plant survival because it can reduce hypoxic stress.

There are two types of aerenchyma, namely primary and secondary aerenchyma. The primary aerenchyma forms in primary tissue and is further classified into two types depending on its formation as lysigenous and schizogenous aerenchyma^14,15^. The lysigenous aerenchyma is formed as a result of programmed cell death^16,17^, while schizogenous aerenchyma is formed as a result of cell separation and differential cell expansion^18^. Secondary aerenchyma is a tissue of secondary origin and differentiates from the phellogen (cork cambium), cambium, or pericycle, all of which can produce either a porous secondary cortex or an aerenchymatous phellem in stems, hypocotyls, and roots^14,18–21^. In woody plant species, the functions of the secondary aerenchyma become more important when the cortex collapses from secondary thickening^22^. Since the development of primary and secondary aerenchyma can be easily observed in the roots, the adventitious roots were primarily used in this study. Previous studies on the aerenchyma have been focused on vegetal plants, and morphological and functional studies on aerenchyma formation of woody plants are still needed.

Some studies have elucidated oxygen dynamics in roots and shoots grown in hypoxic conditions using a Clark-type oxygen microelectrode. For example, Colmer and Pedersen^23^ evaluated the oxygen dynamics in the root and shoot of submerged rice, and the internal oxygen transport in várzea tree species was examined by^24^. In these previous studies, the oxygen concentration was measured in the primary aerenchyma in the roots, and most studies focused on the movement of oxygen according to the formation of primary aerenchyma. Studies on internal pathways for oxygen transport to root cells based on the formation of primary and secondary aerenchyma in one root are limited.^21^. To our knowledge, no study has yet demonstrated changes in oxygen concentration in secondary aerenchyma developed from adventitious roots.

In the adventitious roots of *Syzygium kunstleri* (King) Bahadur and R. C. Gaur grown in hypoxic conditions, differential primary and secondary aerenchyma development was observed within individual roots depending on the age of the tissue. This development may be sensitive to cortex collapse in the root base, which can restrict the function of primary aerenchyma as an oxygen transportation pathway^25^. Therefore, to understand the low-oxygen adaptation mechanism of *S. kunstleri* after cortex collapse near the root base, it is important to characterize the structural continuity of the aerenchyma as well as the way oxygen transport pathways are maintained in primary and secondary aerenchyma.

Since species growing in flooding environments may undergo several morphological and physiological changes, we studied the morphological, anatomical, and physiological effects of flooding on *S. kunstleri* woody plants. We investigated how oxygen is transported to the roots as a phenoplastic reaction, and how transport between primary and secondary aerenchyma is established due to cortex collapse in hypoxic environments over time. For this, we cultivated *S. kunstleri* woody plants over a 50-, 100- and 150-day period in flood conditions, and subsequent developed adventitious roots that formed primary and secondary aerenchyma. We also determined whether tissues that replace the function of primary aerenchyma as an internal oxygen pathway can be identified as secondary aerenchyma, in adventitious roots under long-term hypoxic conditions.

## Results

### Oxygen transport in immature adventitious roots

**Figure 1:**
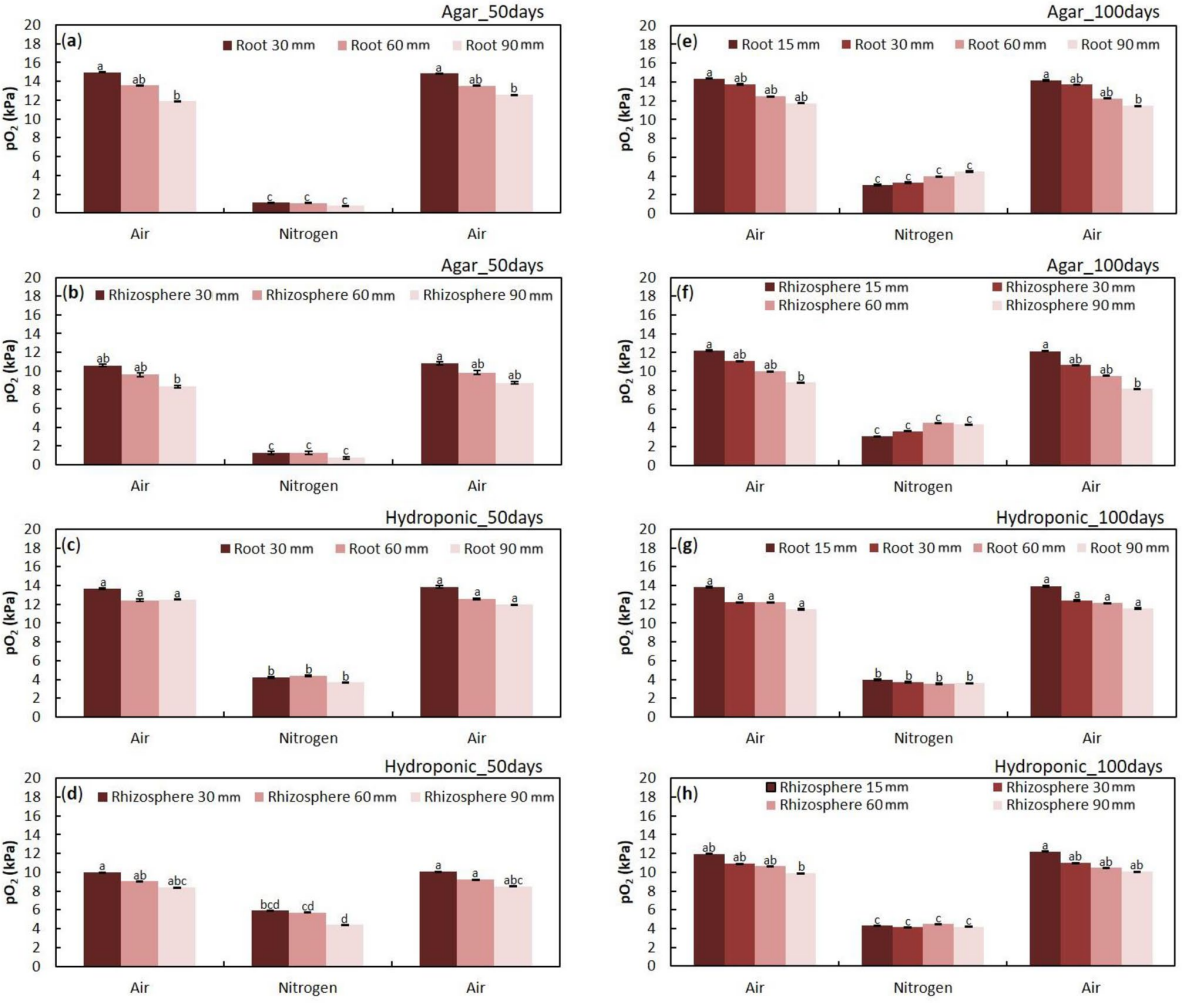
Changes in oxygen partial pressures in the root and rhizosphere after 50 and 100 days of treatment on hydroponic and agar media. The pO_2_ was measured at four different positions (15, 30, 60, and 90 mm from the root-shoot junction). N_2_ was injected for 30 min, then air was injected for 60 min. Values presented are the average of the last minute before the treatment was changed. The pO_2_ in (**a**) the roots and (**b**) rhizosphere of transplants grown in agar medium for 50 days. (**c**) pO_2_ in roots grown in hydroponic medium for 50 days. The pO_2_ in (**d**) the root surface of transplants grown in hydroponic medium for 50 days. The pO_2_ in (**e**) the roots and (**f**) rhizosphere of transplants grown in agar medium for 100 days. (**g**) pO_2_ in roots grown in hydroponic medium for 100 days. The pO_2_ in at (**h**) the root surface of transplants grown in hydroponic medium for 100 days. Data are shown as Means ± standard deviations (*n* = 6–7 roots from six individual plants, average of the last minute before changing the treatments). The same letter indicates no significant difference between measurements (*p* < 0.05, one-way ANOVA and then Tukey’s test for multiple comparisons).

Root and rhizosphere partial pressure of oxygen (pO_2_) were the highest near the root base and decreased acropetally (Fig. 1a–h). The root pO_2_ was higher than rhizosphere pO_2_ in four measuring positions (Fig. 1a–h). The results of pO_2_ measurements from adventitious roots grown in agar and hydroponic media were similar (Fig. 1a–h). Injection of N_2_ or air into stems 0–3 cm above the water level affected pO_2_ in all positions of 15, 30, 60, or 90 mm (Fig. 1a–h). Injection of N_2_ or air into stems 0–3 cm above the water level caused a decrease or increase of pO_2_, respectively, regardless of the root position (Fig. 1a–h). This result was obtained in agar and hydroponic media treatments, indicating that primary aerenchyma developed after 50 and 100 days of growth in hydroponic and agar media and functioned as an oxygen transportation pathway in adventitious roots.

### Oxygen transport in mature adventitious roots

#### Stem secondary aerenchyma/root primary aerenchyma

**Figure 2:**
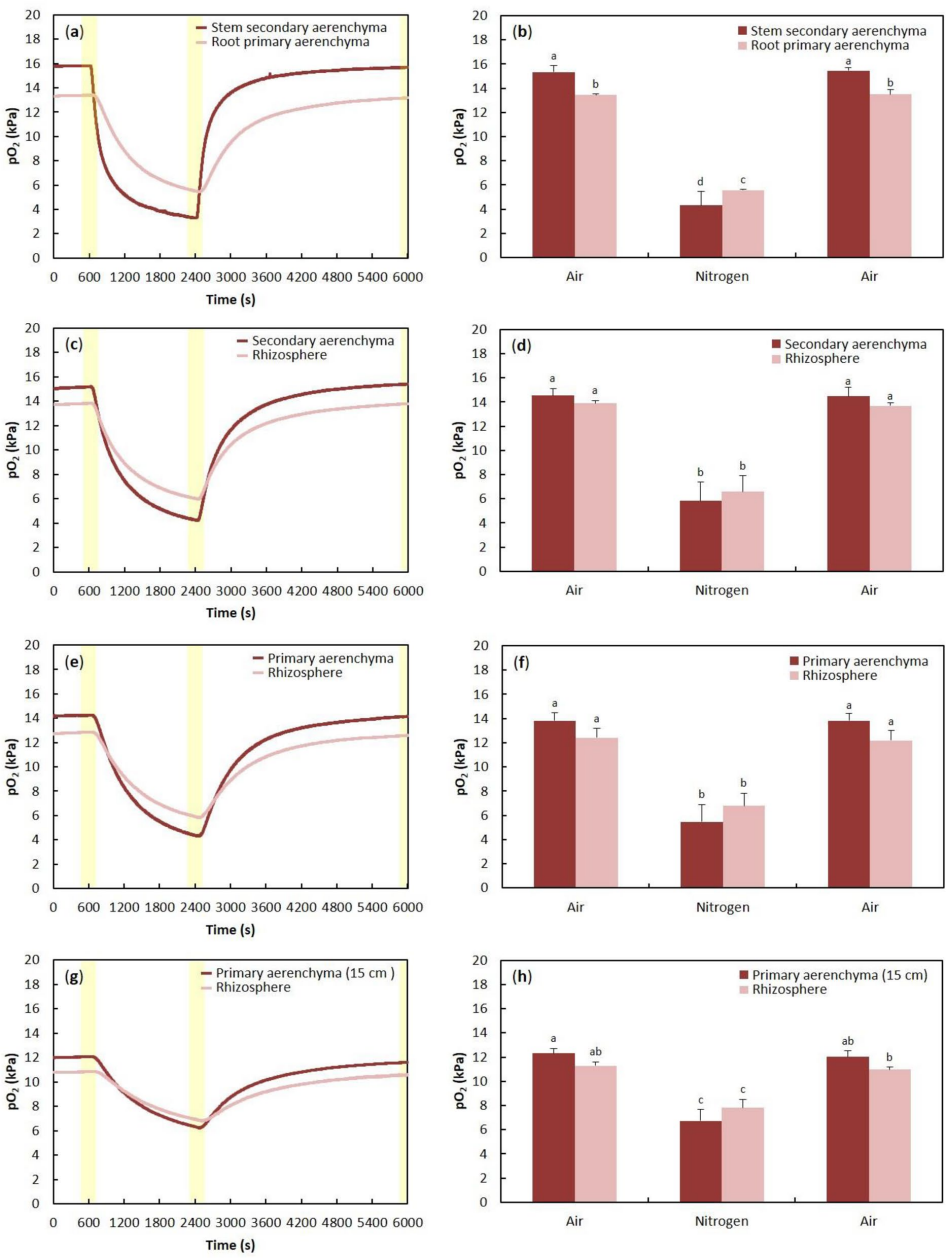
Changes in oxygen partial pressure in stem secondary aerenchyma and root primary aerenchyma (**a**, **b**), primary aerenchyma after cortex collapse and rhizosphere (**c**, **d**), secondary aerenchyma and rhizosphere near the root base (**e**, **f**), and primary aerenchyma in intact cortex and rhizosphere (**g**, **h**) after 150 days of treatment in agar media. (**a**) Time-trace of pO_2_ in stem secondary aerenchyma and root primary aerenchyma. N_2_ was injected instead of air into the stem 0–3 cm above the water level from 2400 to 6000 s. Air was injected from 0 to 600 s and from 2400 to 6000 s. (**c**) Time-trace of pO_2_ in primary aerenchyma and at the root surface. The pO_2_ in primary aerenchyma and root surface (rhizosphere) was measured near a cortex collapse. (**e**) Time-trace of pO_2_ in secondary aerenchyma and rhizosphere. The collapsed cortex was removed from near the root base. (**g**) Time-trace of pO_2_ in primary aerenchyma and at the rhizosphere. The pO_2_ in the primary aerenchyma and rhizosphere was measured 15 cm from the root-shoot junction. (**b**, **d**, **f**, **h**) Values from the last minute (60 records) before changing the treatment (600, 2400, and 6000 s; yellow areas in **a**, **c**, **e**, **g**) were averaged and presented as means ± standard deviations (*n* = 4–6 roots from 4 to 6 individual plants, average of the last minute before changing the treatments). The same letter indicates no significant difference between the treatments at the same measurement area (*p* < 0.05, Bonferroni corrected Mann–Whitney pairwise comparisons, one-way ANOVA, and then Tukey’s test for multiple comparisons). The values are relative and are compared against the initial value (15.34 kPa) of the stem secondary aerenchyma.

The pO_2_ in stem secondary aerenchyma was higher than the pO_2_ in primary aerenchyma in adventitious roots (Fig. 2a,b). The pO_2_ in secondary aerenchyma in the stem and primary aerenchyma in the root decreased or increased when N_2_ or air was injected into the stem base, respectively (Fig. 2a,b). The pO_2_ changed first in secondary aerenchyma in the stem and then in primary aerenchyma in the adventitious root.

#### Root secondary aerenchyma near the root base

**Figure 3:**
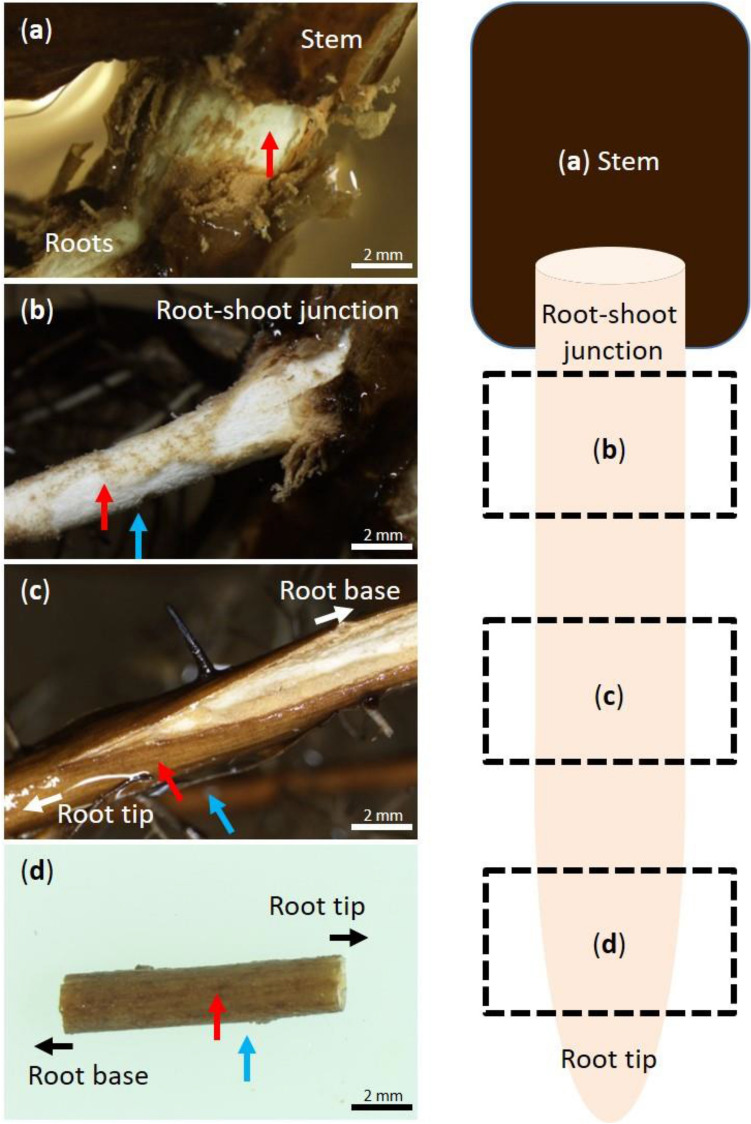
Measurement of oxygen concentration in primary and secondary aerenchyma in stems and adventitious roots after 150 days of hypoxic treatment. The red and blue arrow in each panel indicate the measurement point for the aerenchyma and rhizosphere, respectively. The red arrows indicate the secondary aerenchyma that developed in the (**a**) stem, (**b**) the measurement points of secondary aerenchyma in the root base where the cortex was removed, or (**c**) the primary aerenchyma in the root where cortex collapse had occurred. (**d**) Primary aerenchyma in the root 15 cm from the root-shoot junction.

To examine the continuity between the secondary aerenchyma formed in adventitious roots and the stem, oxygen electrodes were positioned in the secondary aerenchyma and the rhizosphere simultaneously near the root base (Fig. 3b). As a result of the N_2_ or air injection, a decrease or increase in pO_2_ was observed in the secondary aerenchyma and rhizosphere, respectively. A time lag of the pO_2_ change after air or N_2_ injection was observed between the secondary aerenchyma and rhizosphere. When N_2_ or air was injected into the stem, the pO_2_ value of the rhizosphere changed after that of the secondary aerenchyma (Fig. 2c). When air was injected into the stem, the pO_2_ in the secondary aerenchyma was higher than that of the rhizosphere. The N_2_ injection induced larger decreases in pO_2_ in the secondary aerenchyma than the rhizosphere and thus, the pO_2_ in the rhizosphere was higher (Fig. 2c,d). Secondary aerenchyma functions as an oxygen transportation pathway and there were no significant differences between pO_2_ in the secondary aerenchyma and the rhizosphere, suggesting that oxygen is transported from the secondary aerenchyma to the rhizosphere.

#### Root primary aerenchyma with collapsed cortex

To clarify the continuity between the primary aerenchyma and secondary aerenchyma that developed near the collapsed cortex in adventitious roots, the oxygen electrodes were placed in the primary aerenchyma and the rhizosphere, approximately 10 cm from the root-shoot junction where the cortex collapse had occurred (Fig. 3c). The N_2_ or air injection induced a decrease or increase of pO_2_ in both the primary aerenchyma and rhizosphere. When N_2_ or oxygen was injected into the stem base, the value of pO_2_ in the rhizosphere changed later than in the primary aerenchyma (Fig. 2e). When air was injected into the stem base, the pO_2_ in primary aerenchyma was higher than that of the rhizosphere. The pO_2_ in primary aerenchyma was lower than that of the rhizosphere when N_2_ was injected into the stem base (Fig. 2e,f). The primary and secondary aerenchyma were continuous, and there were no significant differences between pO_2_ in the primary aerenchyma and the rhizosphere, indicating that oxygen is transported from the primary aerenchyma of the collapsed cortex to the rhizosphere.

#### Root primary aerenchyma with intact cortex

To elucidate the continuity of primary and secondary aerenchyma formed in adventitious roots, the oxygen electrodes were positioned in the primary aerenchyma and rhizosphere where no cortex collapse occurred, which was approximately 15 cm from the root-shoot junction (Fig. 3d). In this section of the root, no cortex collapse was observed, and the primary aerenchyma remained intact. After an injection of N_2_ or air into the stem 0–3 cm above the water level, a decrease or increase of pO_2_ was observed, respectively, in both the primary aerenchyma and rhizosphere. A time lag was observed between pO_2_ changes in the secondary aerenchyma and rhizosphere after air or N_2_ injection. When N_2_ or oxygen was injected into the stem, the pO_2_ in the rhizosphere changed later than in secondary aerenchyma (Fig. 2g). When air was injected into the stem, the pO_2_ in the primary aerenchyma was higher than that measured in the rhizosphere. The pO_2_ in the primary aerenchyma 15 cm from the root-shoot junction was lower than that in the rhizosphere when N_2_ was injected into the stem base (Fig. 2g,h). The primary and secondary aerenchyma were continuous, and the effect of oxygen from the atmosphere was less at the root tip than at the root base.

### Effects on oxygen transport in root after secondary aerenchyma removal

**Figure 4:**
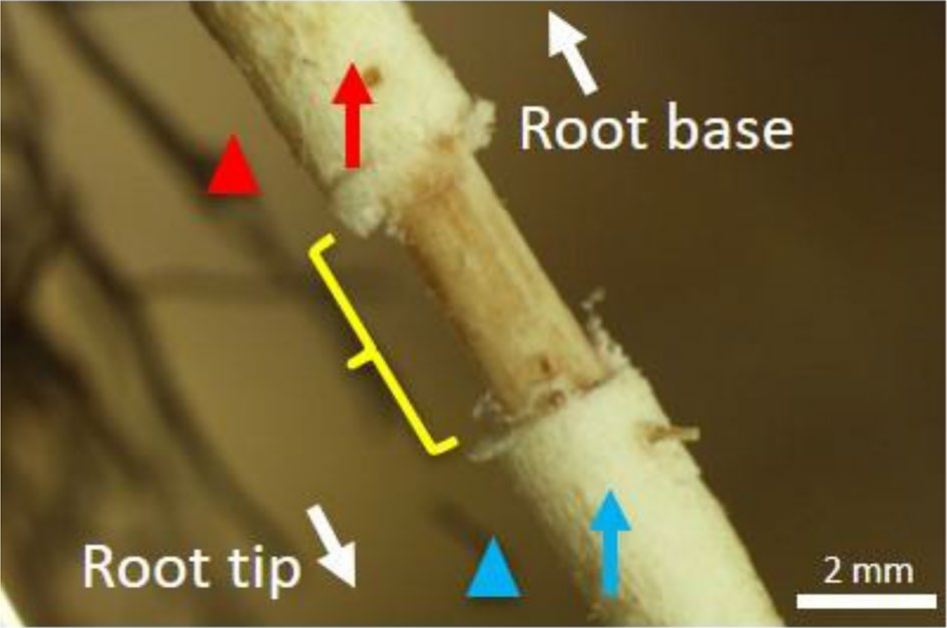
Measurement of oxygen concentrations in remaining portions of secondary aerenchyma in adventitious roots after secondary aerenchyma removal. Adventitious roots were used for measurement of changes of pO_2_ in primary aerenchyma, secondary aerenchyma, and rhizosphere after 150 days of treatment in agar media. Yellow parentheses show where the secondary aerenchyma was removed from the adventitious root. Oxygen concentration was measured in the secondary aerenchyma (red and blue arrow) above and below where the secondary aerenchyma had been removed. Oxygen concentration was measured at the root surface (rhizosphere) above and below where the secondary aerenchyma had been removed from the root (red and blue arrowheads). White arrows indicate the orientation of the root.

**Figure 5:**
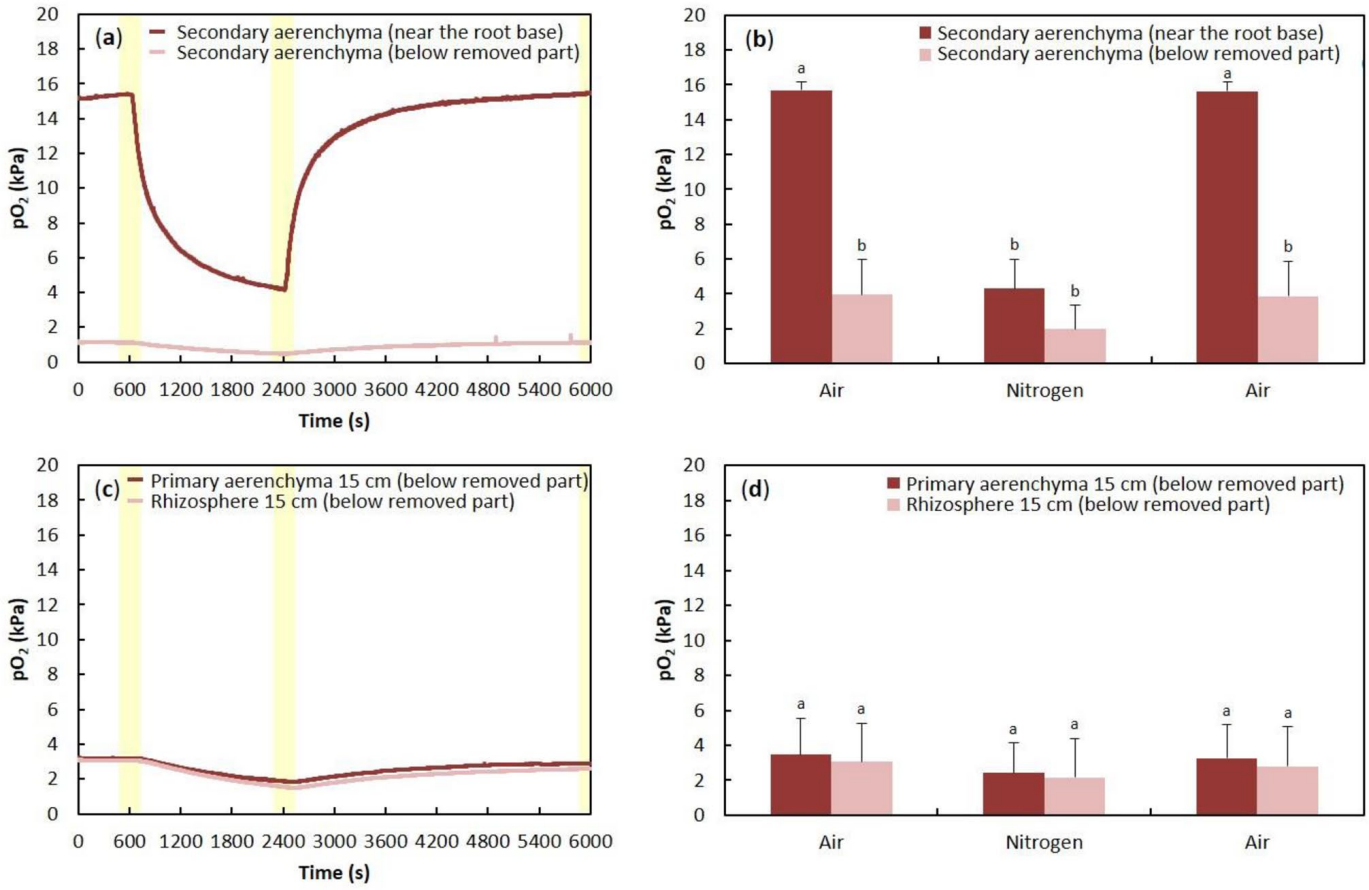
Changes in oxygen partial pressure above and below secondary aerenchyma that was removed 150 days after treatment in agar media (**a**, **b**). Changes in oxygen partial pressure in primary aerenchyma with an intact cortex and rhizosphere after secondary aerenchyma removal 150 days after treatment in agar media (**c**, **d**). Secondary aerenchyma was removed near the root base (Fig. 4). (**a**) Time-trace of pO_2_ in secondary aerenchyma at two different positions above and below where secondary aerenchyma had been removed from the root. N_2_ was injected in exchange for air into the stem 0–3 cm above the water level from 2400 to 6000 s. Air was injected from 0 to 600 s and from 2400 to 6000 s. (**c**) Time-trace of pO_2_ in primary aerenchyma and the rhizosphere. The pO_2_ in the primary aerenchyma and at the root surface (rhizosphere) was measured approximately 15 cm from the root-shoot junction after the secondary aerenchyma was removed. (**b**, **d**) The average of the last one minute (60 records) before changing the treatments (600, 2400, and 6000 s; yellow areas in **a**, **c**) are expressed as the means ± standard deviations (*n* = 6 roots from six individual plants, average of the last minute before changing the treatments). The values are relative and are compared against the initial value (15.34 kPa) of the stem secondary aerenchyma. Similar letter indicates no significant difference between measurements within the same area (*p* < 0.05, one-way ANOVA, and then Tukey’s test for multiple comparisons).

To examine the oxygen transport function of the secondary aerenchyma in adventitious roots, the secondary aerenchyma was removed near the root base, and oxygen electrodes were placed on the remaining secondary aerenchyma remaining on either side of the removed section (Fig. 4). After injection of N_2_ or air to the stem base, the secondary aerenchyma near the root-shoot junction showed approximately 15–16 kPa when air was injected and 4 kPa when N_2_ was injected (Fig. 5a,b). In secondary aerenchyma farther away from the root-shoot junction and to the other side of where the secondary aerenchyma had been removed, there was no response to air or N_2_ injection (Fig. 5a,b). The patterns of pO_2_ in the rhizosphere after air or N_2_ injections were similar to those found in the secondary aerenchyma on both sides of the removed section (data not shown).

### Effects on oxygen transport in the primary aerenchyma after secondary aerenchyma removal

After removing the secondary aerenchyma near the root base (Fig. 4), the oxygen electrodes were positioned in the primary aerenchyma and rhizosphere in a portion of the root without cortex collapse, approximately 15 cm from the root-shoot junction. After injection of N_2_ or air into the stem 0–3 cm above the water level, the pO_2_ values were maintained at low levels. The injection of N_2_ or air after removing the secondary aerenchyma near the root base did not affect the pO_2_ in primary aerenchyma near the root tip (Fig. 5c,d). Additionally, the pO_2_ in the root and rhizosphere were similar both before and after the injection of N_2_ or air (Fig. 5c,d).

## Discussion

The oxygen transport in primary aerenchyma of adventitious roots was confirmed by a series of pO_2_ measurements at different positions along the root. As a result of measuring pO_2_ in primary aerenchyma, we observed that the pO_2_ was highest in the root base and lower toward the root tip, regardless of the age of the root (Fig. 1). Moreover, pO_2_ in primary aerenchyma was unaffected near the root base and was not significantly different between roots regardless of the development of secondary aerenchyma, which was determined by the treatment period (Fig. 1). The decrease of pO_2_ in the primary aerenchyma was observed after injecting N_2_ into the stem 0–3 cm above the water level (Fig. 1) and confirmed that the primary aerenchyma in adventitious roots was connected to the aerial stem. Also, root pO_2_ was highest near the root base, and acropetally decreased (Fig. 1). This difference in pO_2_ in the roots confirmed that oxygen was transferred from the root base to the root tip. The difference of pO_2_ in the roots may also cause a difference of pO_2_ at the root surface.

A similar pattern of pO_2_ change was found at the root surface (rhizosphere) as well as in the root (Fig. 1), which is consistent with reports in *Salix martiana* and *Tabernaemontana juruana*^24^. However, in rice^26–28^ and maize^2^, the radial oxygen loss (ROL) was the highest in the root tip and the lowest in the root base due to a barrier at the root base that suppressed ROL^2,28,29^. Our results suggest an absence of ROL barrier in the cortex where the primary aerenchyma is developed; therefore, further anatomical and physiological studies are warranted to analyze the development of the suberin layer in the cortex. Despite the absence of an ROL barrier, which seems to have no harmful and fatal effects on plants and roots, oxygen is sufficiently transported to the root’s tip, possibly because the primary and secondary aerenchyma are connected and serve as an oxygen pathway. It is assumed that ROL occurs in areas other than the root tip, reducing the inflow of methane, hydrogen sulfide, and other gases into the aerenchyma. Through the ROL, oxygen can react with chemically reduced elements present in the soil, such as iron, which reacts with oxygen to form an iron plaque on the root surface. This plaque plays a vital role in controlling the sequestration of excess loads of nutrients and contaminants in wetlands^30^ as the oxygen leakage oxidizes and detoxifies potentially harmful reducing substances in the rhizosphere. In general, the oxygen consumption of microorganisms is related to the presence of rhizospheric oxygen. Thus, oxygen supply and the related redox potential (E_H_) are important parameters for interactions between roots and microorganisms in the rhizosphere^31^. In *Vallisneria spiralis*, rhizospheric oxygen was related to rhizosphere physicochemical parameters and the microbial community^32^. The presence or absence of the ROL barrier can, therefore, affect plant growth and adaptation to hypoxic conditions.

The oxygen profiles of primary and secondary aerenchyma were investigated by measuring changes in root pO_2_ after injecting N_2_ or air into the stem 0–3 cm above the water level. We observed that the pO_2_ in the primary aerenchyma decreased or increased when N_2_ or air was injected into the stem, respectively (Fig. 2a,b,e–h). When the cortex at the root base was removed, changes in pO_2_ in primary and secondary aerenchyma were still observed (Fig. 2c–h). Moreover, this change in pO_2_ was also observed in the primary aerenchyma near a cortex collapse (Fig. 2e,f). These results show that primary and secondary aerenchyma function as an oxygen transport pathway. Although there was no primary aerenchyma in the root base, changes in pO_2_ were observed in primary aerenchyma located near the root tip. It is hypothesized that the primary and secondary aerenchyma are connected in the roots and transport oxygen from the root base to the root tip.

We also confirmed that the primary aerenchyma in the root is connected to the secondary aerenchyma formed in the stem (Fig. 2). The continuity of the aerenchyma between the stem and roots was confirmed by the transfer of oxygen into the roots through the secondary aerenchyma formed in the stem of soybean^21^. Soybean is a model species used in the study of the formation of secondary aerenchyma^20,21,33^. Secondary aerenchyma forms in the soybean stem and serves as a 'snorkel' that enables oxygen transport from the air to submerged roots^21^. However, our work presented here is the first study to demonstrate the development, function, and connectivity of primary and secondary aerenchyma within an adventitious root. Further, we show that primary and secondary aerenchyma are linked by measuring changes in pO_2_ through the stem and root tissue, as well as showing that the connection between primary and secondary aerenchyma could be demonstrated using anatomical studies.

So far, the development of secondary aerenchyma has been observed only in a limited number of herbaceous and woody plants like *Glycine max*, *Sesbania cannabina*, *Macairea radula*, and *Lavoisiera bergii*^20,34,35^. Secondary aerenchyma was also observed in several forms but was mostly limited to anatomical approaches. Secondary aerenchyma in soybean was seen to arise from cell divisions in the pericycle. The entire secondary aerenchyma in soybean consisted only of cells resulting from that process and formed as white spongy tissue^25,36^. The secondary aerenchyma of *S. kunstleri* also developed by cell division but, in contrast to soybean, the repeated structure formed by the layers of phellem cells and the division of elongated cells between the layers of phellem cells seem to be a distinctive process of secondary aerenchyma formation in *S. kunstleri*^25^. To our knowledge, this is the first study to measure changes in oxygen concentration directly in secondary aerenchyma and to confirm the oxygen transport function of secondary aerenchyma in stems and roots.

**Figure 6:**
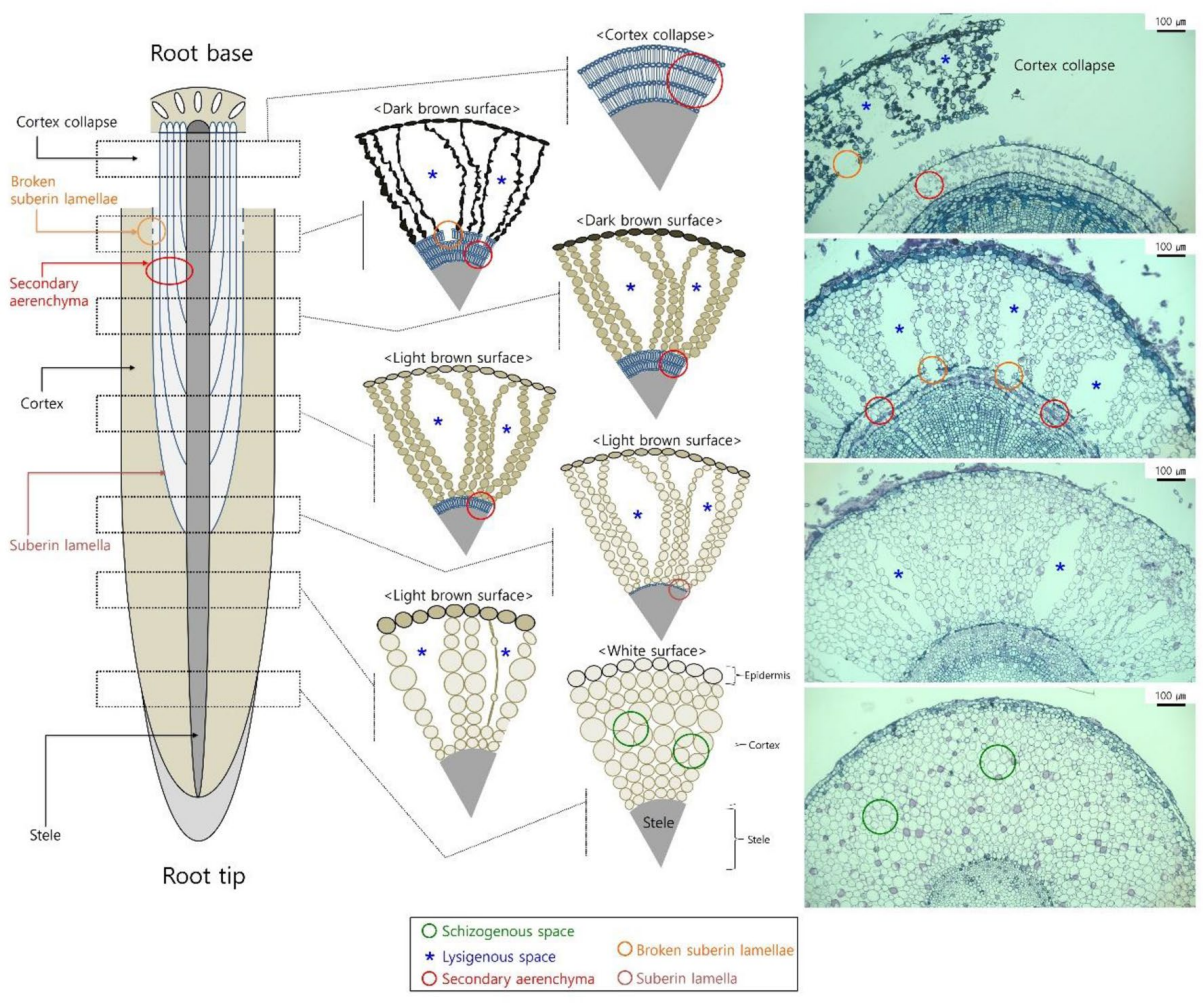
Diagram of the primary and secondary aerenchyma development sequence along the *S. kunstleri* adventitious roots grown in agar media for 150 days. Primary aerenchyma is root cortex tissue with large intercellular spaces. Primary aerenchyma forms through two distinct development processes: separation of cells (schizogenous aerenchyma) or cell death (lysigenous aerenchyma). Schizogenous aerenchyma develops only near the root tip. Lysigenous aerenchyma develops in the middle part and near the root base. Secondary aerenchyma differentiates from the phellogen, cambium, and pericycle of stems or roots. Secondary aerenchyma is composed of suberin lamellae and begins to develop inside the lysigenous aerenchyma. Secondary aerenchyma develops after the primary aerenchyma has developed.

In *S. kunstleri*, secondary aerenchyma was observed in the hypertrophied stem after flooding treatment (Fig. 3), similar to what has been reported in soybean^20^. Multiple layers were formed inside the cortex in *S. kunstleri* adventitious roots (Fig. 6), which were a different structure from the secondary aerenchyma in soybean^20,25^. The secondary aerenchyma formed from multiple layers that could sufficiently transport oxygen even when the primary aerenchyma was collapsed or completely removed (Fig. 2c,d). The oxygen transport role of secondary aerenchyma was clearly demonstrated by the reduction of oxygen concentration in the secondary aerenchyma and primary aerenchyma below it after the secondary aerenchyma had been removed near the root base (Fig. 5a–d). Although the transport of oxygen in the longitudinal direction of secondary aerenchyma is clearly shown in this work, additional anatomical studies on oxygen transport from the secondary aerenchyma to the adjacent rhizosphere, that is, radial oxygen movement, remain warranted. If the ROL from primary and secondary aerenchyma is investigated in greater depth, the rate and efficiency of oxygen transport in primary and secondary aerenchyma can be calculated, and the ability of *S. kunstleri* to adapt to low-oxygen conditions can be further clarified. If these factors are better understood, it is believed that studies on *S. kunstleri* as a reforestation tree species in a low-oxygen environment, such as peat swamp forest in Thailand, can be conducted more effectively.

The development of primary and secondary aerenchyma is correlated with flooding stress^20,25,35,36^. The oxygen concentration in agar media is lower than in hydroponic media^37^ and thus, the anatomical difference of aerenchyma development could be influenced by the oxygen concentration of the growth medium^25^. However, there was no difference in function between primary and secondary aerenchyma that is dependent on medium oxygen concentration. As the aerenchyma was more developed in the roots that developed after 100 days compared to those developing after 50 days, it is assumed that oxygen supply to the root tip is much smoother. In addition, as aerenchyma develops further in the roots of agar medium, pO_2_ in this root tip is assumed to be higher than pO_2_ in root tips grown using hydroponic medium and supplied with N_2_. Aerenchyma development may serve as a buffer in hypoxic conditions, allowing it to be beneficial in flooded conditions.

Many flood-tolerant species develop aerenchyma to transport oxygen from the stem to the roots^12,38–41^. Woody plants undergo cortex collapse in the roots during the secondary thickening of cells^22^. Thus, under hypoxic conditions, the development of secondary aerenchyma and the continuity of the primary and secondary aerenchyma, play a critical role in the growth and structure of woody plants. In *S. kunstleri*, after the development of primary aerenchyma in the cortex, the primary aerenchyma is destroyed by the collapse of the cortex. However, multiple layers of secondary aerenchyma develop inside the cortex while the primary aerenchyma is present (Fig. 6)^25^. Therefore, secondary aerenchyma is needed to support or replace the primary aerenchyma.

The secondary aerenchyma layer on the roots of *S. kunstleri* grown in hypoxic environment comprises suberin layers^25^, which limit the transport of oxygen. The continuous between primary and secondary aerenchyma was observed in the cross section of the root where they develop (Fig. 6). The outermost layer of the secondary aerenchyma adhered to the primary aerenchyma might have collapsed thereby creating a pathway between primary and secondary aerenchyma where molecules in gaseous forms can be transported. Although various anatomical pathways between primary and secondary aerenchyma may exist, the collapse of the secondary aerenchyma layer was a clearly observed pathway in this study. When primary and secondary aerenchyma develop in the same position on the root, oxygen can freely move from the primary to secondary aerenchyma. The mechanism underlaying the connection between the two aerenchyma warrants investigation. Furthermore, anatomical studies on the mechanisms by which oxygen passes through the cells of aerenchyma are also warranted.

Studies of the oxygen transport function of primary aerenchyma have identified the relationship between aerenchyma development and oxygen transport function, as well as characterized the changes in oxygen concentration in the primary aerenchyma of the roots in *Oryza sativa*^23^, *Phragmites australis*^42^, *Rumex palustris*, and *R. acetosa*^43^, and várzea tree species^24^. Both primary and secondary aerenchyma develops in adventitious roots of *S. kunstleri,* and the oxygen transport functions of these aerenchymas were clarified in this study. In addition, through the measurement of the developmental process of primary and secondary aerenchyma and changes of pO_2_ in these tissues, we show that secondary aerenchyma can support the role of primary aerenchyma and take over this role in the long term. In conclusion, we have illustrated the oxygen transport function of primary and secondary aerenchyma in the adventitious roots of woody plants. This study will contribute to the understanding of the adaptation mechanism to environmental changes caused by climate change in woody plants.

## Methods

### Plant materials

*Syzygium kunstleri* was used for all experiments. Experiments were conducted with 6- to 12-month-old cuttings of *S. kunstleri* derived from 3- to 4-year-old saplings grown from seeds collected from a wetland in Narathiwat Province, Thailand. Cuttings were rooted in a pot (60 × 26 × 27 cm) containing Akadama soil (granular loamy soil) for about 6 months under well-watered conditions in a phytotron under natural light at the University of Tokyo, Japan. Light intensities varied between 1000 and 2000 μmol m^-2^ s^-1^ (PAR); day length was approximately 12 h. Temperature varied in 12 h cycles between 30 °C in the day and 25 °C during the night. After rooting, the cuttings were transferred to round 7 (diameter) × 20 (height) cm pots filled with quartz sand, with each pot containing one cutting.

After washing the roots with tap water, the 10–15-cm tall cuttings were transferred to either hydroponic or agar-solidified (0.1% w/v) media in a container (17 × 25 × 24 cm). Six cuttings (10–15 cm) were transferred to each container, with six containers for each treatment. The nutrient solution contained the following: 4 mM NH_4_NO_3_, 0.6 mM NaH_2_PO_4_, 0.6 mM KCl, 0.35 mM CaCl_2_, 0.25 mM MgSO_4_, 10 μM FeSO_4_, 20 μM H_3_BO_3_, 2 μM MnCl_2_, 2 μM ZnSO_4_, 2 μM CuSO_4_, and 2 μM Na_2_MoO_4_, at a pH between 5.5 and 6.0^44^. Plants were cultivated in a phytotron. Cuttings were transferred to fresh medium every 2 weeks and grown for 50, 100, and 150 days on hydroponic or agar-solidified media, after which adventitious roots were formed. Six cuttings were grown in each of six containers for each two treatments over three cultivation periods. Sample roots were randomly selected from six cuttings in a container, while roots were sampled from separate cuttings. All measurements were conducted on adventitious roots that were developed after transfer to the hydroponic or agar-solidified media.

### O_2_ microelectrode measurements

For measuring the O_2_ partial pressure (pO_2_), Clark-type O_2_ microelectrodes with gold-tipped cathodes were prepared as described by^45^. Microelectrodes were connected to a two-channel picoammeter (PA2000, Unisense A/S, Aarhus, Denmark), and the signal was converted with an analog-to-digital converter. This signal was logged into a computer through a data acquisition program (PicoLog for Windows, Pico Technology) every second. The O_2_ microelectrode signals were converted from currents into pO_2_ using individual calibrations of 0–100% of air equilibrium before and after each experiment^46,47^. In this study, the following formula was used to convert the current value (C[x]) measured by the microelectrodes to the oxygen partial pressure (pO_2_[x])

$$ {\text{pO}}_{2} \left[ {\text{x}} \right] = {{\left( {{\text{pO}}_{2} \left[ {{\text{Air}}} \right] - {\text{pO}}_{2} \left[ {{\text{N}}_{2} } \right]} \right)\left( {{\text{C}}\left[ {\text{x}} \right] - {\text{C}}\left[ {{\text{N}}_{2} } \right]} \right)} \mathord{\left/ {\vphantom {{\left( {{\text{pO}}_{2} \left[ {{\text{Air}}} \right] - {\text{pO}}_{2} \left[ {{\text{N}}_{2} } \right]} \right)\left( {{\text{C}}\left[ {\text{x}} \right] - {\text{C}}\left[ {{\text{N}}_{2} } \right]} \right)} {\left( {{\text{C}}\left[ {{\text{Air}}} \right] - {\text{C}}\left[ {{\text{N}}_{2} } \right]} \right)}}} \right. \kern-\nulldelimiterspace} {\left( {{\text{C}}\left[ {{\text{Air}}} \right] - {\text{C}}\left[ {{\text{N}}_{2} } \right]} \right)}} $$

where pO_2_ is oxygen partial pressure, and C is the current value measured by the microelectrode. The oxygen partial pressure in N_2_ (pO_2_[N_2_]) and air (pO_2_[Air]) at 30 °C was 0 kPa and 20.9 kPa, respectively.

**Figure 7:**
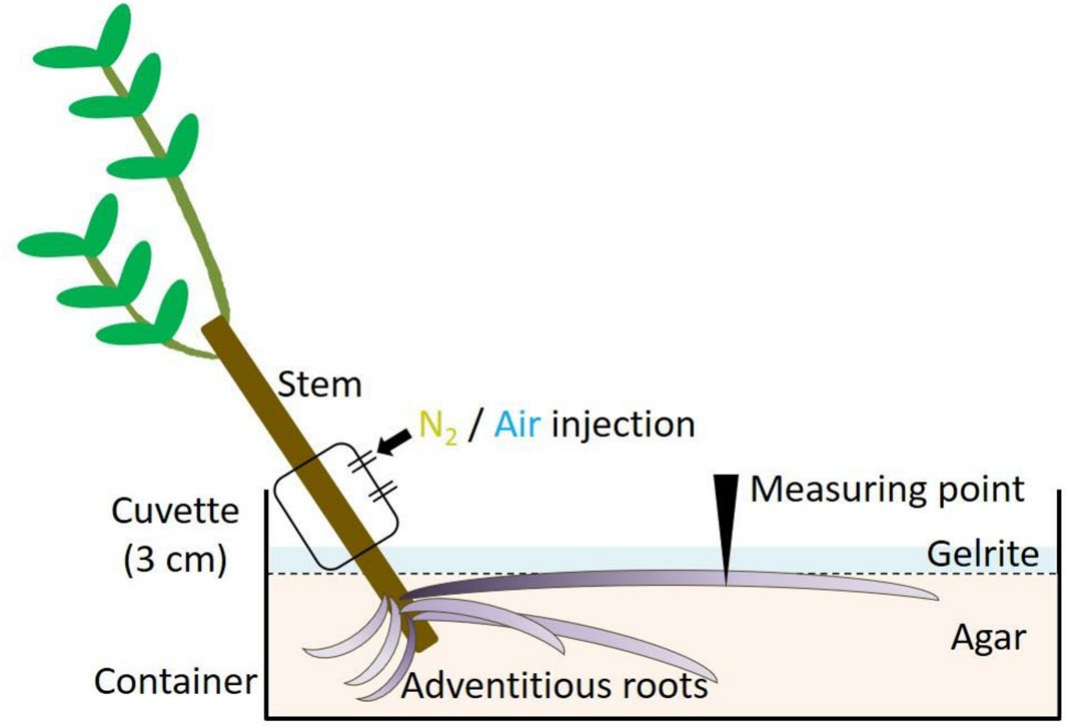
Diagram of how oxygen partial pressure was measured in adventitious roots. Root systems were fixed in containers if deoxygenated solid agar (1.5% w/v) at the same water level at which they were grown in hydroponic or agar media. The target adventitious roots were fixed near the surface of the solid agar and covered with deoxygenated solid gelrite (2% w/v). Air or N_2_ was injected through a cuvette that covered the stem. Positioning of the microelectrode inside the root was aided with a microscope and adjusted with a boom stand.

For each experiment, cuttings were transferred to a container filled with deoxygenated solid agar (1.5% w/v) at room temperature (25–27 °C). Root systems were fixed in the container at the same water level in both hydroponic and agar media conditions. The target adventitious roots were fixed near the surface of the solid agar and covered with deoxygenated solid gelrite (2% w/v) in a layer approximately 5 mm thick to assist microscopic imaging (Fig. 7). Using a micromanipulator, a Clark-type O_2_ microelectrode was placed in the primary aerenchyma, secondary aerenchyma, and the rhizosphere, located approximately 100 μm inside and outside the root surface. A microscope and boom stand were used to assist positioning of the microelectrode inside the root cortex.

Adventitious roots that developed after 50 and 100 days of growth in hydroponic and agar media were used to examine the pO_2_ in primary aerenchyma. Root and rhizosphere pO_2_ changes were measured at 15, 30, 60, and 90 mm from the root-shoot junction. The adventitious roots that developed after 150 days of growth in agar media were used to examine the continuity between primary and secondary aerenchyma. The oxygen electrodes were simultaneously positioned at the secondary aerenchyma in stems and primary aerenchyma in adventitious roots (Fig. 3a,d). The changes of pO_2_ at the two positions were then measured concomitantly following N_2_ or air injection into stems 0–3 cm above the water level. To measure the function and continuity of primary and secondary aerenchyma, the cortex of the root base was removed with a razor blade to expose the secondary aerenchyma. The length of the exposed secondary aerenchyma was approximately 4 cm from the root-shoot junction (Fig. 3b). Oxygen microelectrodes were positioned in the primary aerenchyma, secondary aerenchyma, and rhizosphere of the root (Fig. 3b–d). Air and N_2_ were alternately injected through the cuvette at intervals of 30 min on the 0–3 cm of stem above water level. Then, changes of pO_2_ in primary aerenchyma, secondary aerenchyma, and rhizosphere were measured (Fig. 3a–d). Changes in pO_2_ was also measured after removal of the secondary aerenchyma near the root base (Fig. 4).

### Statistics

The N_2_ was injected in exchange for air in the stem 0–3 cm above the water level and a time-trace of pO_2_ was measured in primary aerenchyma, secondary aerenchyma, and at the root surface (rhizosphere). The measurements were recorded for an average of 1 min before changing treatment. To test the difference in those values among the different areas (i.e., primary aerenchyma, secondary aerenchyma, rhizosphere) and condition (i.e., air injection or N_2_ injection), a Bonferroni corrected Mann–Whitney pairwise comparison was conducted followed by a Kruskal–Wallis test (*p* < 0.05) and one-way ANOVA followed by Tukey’s test (*p* < 0.05) for multiple comparisons. All data are presented as mean ± standard error. Analyses were carried out using SPSS Statistics Version 25 software (IBM Software, Armonk, NY, USA).

## Data Availability

No datasets were generated or analyzed during the current study.
